# Long-Term Protection against Diphtheria in the Netherlands after 50 Years of Vaccination: Results from a Seroepidemiological Study

**DOI:** 10.1371/journal.pone.0148605

**Published:** 2016-02-10

**Authors:** E. M. Swart, P. G. M. van Gageldonk, H. E. de Melker, F. R. van der Klis, G. A. M. Berbers, L. Mollema

**Affiliations:** Centre for Infectious Disease Control, National Institute for Public Health and the Environment, Bilthoven, the Netherlands; University of Cambridge, UNITED KINGDOM

## Abstract

**Background and Aims:**

To evaluate the National Immunisation Programme (NIP) a population-based cross-sectional seroepidemiological study was performed in the Netherlands. We assessed diphtheria antitoxin levels in the general Dutch population and in low vaccination coverage (LVC) areas where a relatively high proportion of orthodox Protestants live who decline vaccination based on religious grounds. Results were compared with a nationwide seroepidemiological study performed 11 years earlier.

**Methods:**

In 2006/2007 a national serum bank was established. Blood samples were tested for diphtheria antitoxin IgG concentrations using a multiplex immunoassay for 6383 participants from the national sample (NS) and 1518 participants from LVC municipalities. A cut-off above 0.01 international units per ml (IU/ml) was used as minimum protective level.

**Results:**

In the NS 91% of the population had antibody levels above 0.01 IU/ml compared to 88% in the 1995/1996 serosurvey (p<0.05). On average, 82% (vs. 78% in the 1995/1996 serosurvey, p<0.05) of individuals from the NS born before introduction of diphtheria vaccination in the NIP and 46% (vs. 37% in the 1995/1996 serosurvey, p = 0.11) of orthodox Protestants living in LVC areas had antibody levels above 0.01 IU/ml. Linear regression analysis among fully immunized individuals (six vaccinations) without evidence of revaccination indicated a continuous decline in antibodies in both serosurveys, but geometric mean antibodies remained well above 0.01 IU/ml in all age groups.

**Conclusions:**

The NIP provides long-term protection against diphtheria, although antibody levels decline after vaccination. As a result of natural waning immunity, a substantial proportion of individuals born before introduction of diphtheria vaccination in the NIP lack adequate levels of diphtheria antibodies. Susceptibility due to lack of vaccination is highest among strictly orthodox Protestants. The potential risk of spread of diphtheria within the geographically clustered orthodox Protestant community after introduction in the Netherlands has not disappeared, despite national long-term high vaccination coverage.

## Introduction

Despite the success of routine vaccination, diphtheria is still a serious child health problem with 5,000 diphtheria cases globally in 2012, occurring in particular in South-East Asia [1]. The major diphtheria outbreak in the Newly Independent States of the former Soviet Union during the 1990s, with > 150,000 cases indicated that diphtheria can reemerge in susceptible populations [2–4]. In the Netherlands, diphtheria was endemic before introduction of diphtheria vaccination in 1957. The last diphtheria epidemic occurred during World War II with > 190,000 cases reported between 1940 and 1945. Since 1960, diphtheria has become a rare disease in the Netherlands [5]. However, the recent diphtheria case in Spain highlights the importance of vaccination against diphtheria, even in non-endemic countries [6]. In addition, an important issue emerging in literature is the shortage of diphtheria antitoxin (DAT) [6–9]. This immunoglobulin preparation is needed for the treatment of diphtheria and most effective when administered as early as possible [6–8]. The possible lack of appropriate DAT supply emphasizes the need of maintaining high vaccination coverage [6].

Vaccination against diphtheria was introduced in the Dutch National Immunization Program (NIP) in 1957 using a combination vaccine including the diphtheria, tetanus and whole-cell pertussis (DTwP) vaccine. From 1962 onwards, infants received a combined vaccine including diphtheria, tetanus, whole-cell pertussis and inactivated polio vaccine (DTwP-IPV) at three, four, and five months of age, followed by a booster vaccination at 11 months of age. Booster vaccinations at four and nine years of age with DT-IPV were added to the NIP in 1965. From 1999 onwards, the first three infant doses were given at two, three and four months of age. The schedule with six diphtheria vaccinations is still in use, however, the combination vaccines used in the NIP in the Netherlands have changed several times in composition and of manufacturer [10]. In 2003 *Haemophilus influenza* (Hib) vaccine was added to the DTwP-IPV vaccine for infants (DTwP-IPV/Hib) and in 2005 the infant whole-cell pertussis vaccine was replaced by an acellular pertussis vaccine (DTaP-IPV/Hib) [11]. In 2006 a seven-valent pneumococcal vaccine conjugated to a non-toxic, fully immunogenic mutant of diphtheria toxin (CRM197) was added to the NIP at two, three, four, and 11 months of age for all children born in or after April 2006. In addition, in July/August 2006, acellular pertussis vaccine was added to the booster combination vaccine for 4-year-olds (DTaP-IPV). Vaccination coverage for diphtheria has been continuously high (> 90%) for at least the last 35 years [12]. However, in the Netherlands there are areas with low vaccination coverage (LVC). In these communities reside a relatively high proportion of socio-geographically clustered orthodox Protestant individuals who decline vaccination based on religious grounds. Vaccination coverage among orthodox Protestant individuals was overall about 60% (measured in 2006/2007 and 2008) [13].

We present results of a national seroepidemiological study performed in 2006/2007 assessing diphtheria antibodies in the Dutch general population as well as in LVC areas where many orthodox Protestants live. We compare our results with the previous national study conducted in 1995/1996 [14]. This enables us to study the impact of potential further natural- and vaccination-induced waning immunity in adults as well as changes in susceptibility in orthodox Protestant individuals. In addition, it enables us to study the impact of changes made in the vaccination schedule.

## Materials and Methods

### Study population and design

From February 2006 through June 2007, a large national serum bank was established by means of a population-based cross-sectional seroepidemiological study (i.e. Pienter2 study). Details on study design and data collection have been described elsewhere [15,16]. In brief, a national sample (NS) was drawn using a two-stage cluster sampling technique. The Netherlands was divided into five geographical regions of approximately equal population size. Within each region, eight municipalities (e.g. clusters) were randomly selected with a probability proportional to their size. An age-stratified sample of 380–500 individuals was drawn randomly from the population register of each of the 40 sampled municipalities. Age strata were 0, 1–4, 5–9, 10–14, …, 75–79 years of age. The youngest two age strata were oversampled because of expected lower response rates. To assess immunity against NIP diseases in migrants separately, oversampling of non-Western migrants was performed in 12 out of 40 municipalities from the NS. To assess immunity in orthodox Protestant individuals who refuse vaccination, eight LVC municipalities were sampled. Approval for this study was obtained from the Medical Ethics Testing Committee of the Foundation of Therapeutic Evaluation of Medicines (METC-STEG) in Almere, the Netherlands (clinical trial number: ISRCTN 20164309). All participants provided signed informed consent prior to participation. Signed informed consent for minors was obtained from two parents or guardians. Participants were requested to donate a blood sample at a clinic, to complete a questionnaire at home, and to bring their vaccination certificates. If the certificates were not available, vaccination status was obtained from the local authority for registration of vaccination.

### Laboratory methods

Serum IgG antibodies directed against diphtheria toxin were analyzed as described previously using a fluorescent microsphere-based multiplex immuno assay (DTaP MIA) [17]. International cut-off standards were used for classification of diphtheria antitoxin antibody concentrations. Antibody levels below 0.01 international units per ml (IU/ml) were considered non-protective, levels of 0.01 IU/ml–0.1 IU/ml were considered to provide basic protection and levels above 0.1 IU/ml were considered to provide full protection against diphtheria [18].

### 1995/1996 serosurvey

The study design and data collection of the seroepidemiological study conducted from October 1995 through December 1996 (i.e. Pienter1 study) was comparable to the 2006/2007 serosurvey and has been described in detail elsewhere [19,20].

In the 1995/1996 serosurvey, the toxin-binding inhibition assay (ToBI) was used to determine diphtheria antibody concentrations, as described previously [21]. To enable a proper bridging between both serosurveys, a randomly selected subsample of 135 samples with a broad range of concentrations from individuals of all age groups from both serosurveys were analyzed in the ToBI and MIA. Concentrations were log-transformed and the Bland-Altman plot demonstrated good agreement between both methods (S1 File). A good correlation was found (R = 0.976) between the ToBI (X) and the MIA (Y) with y = 0.6792x^0.948^ (S1 File). This equation was used to transform all concentrations of the 1995/1996 serosurvey measured with ToBI to make them comparable for both serosurveys. For comparison of geometric mean concentrations (GMCs) between both serosurveys, antibody concentrations below 0.01 IU/ml were set at 0.005 IU/ml using the lower limit of quantitation (LLOQ) of the ToBI and not of the MIA (LLOQ = 0.01 and 0.001 IU/ml, respectively).

We present transformed values of the 1995/1996 serosurvey to enable direct comparison between both serosurveys.

### Statistical analyses

Analyses were performed using SAS, version 9.3 (SAS Institute Inc., Cary, NC, USA) and R [22].

#### Seroprevalence and geometric mean concentration (in NS and LVC)

Seroprevalences and GMCs in the NS were estimated by weighting for age, gender, ethnicity and degree of urbanization to match the Dutch population distribution as to that of 1 January 2007 [23]. Seroprevalence and GMCs in the LVC sample were weighted by age and gender. Adjustment for the two-stage cluster sampling design was done by taking the strata (five regions) and clusters (40 municipalities) into account in all analyses of the NS. In the analyses of the LVC sample the cluster sampling (eight municipalities) was taken into account.

The LVC sample was stratified by vaccination coverage as defined by Ruijs et al. [13]. The first group represented the low (<25%) or intermediate (50–75%) vaccination coverage clusters (i.e. orthodox Protestant individuals). The second group represented the moderate to high (>85%) vaccination coverage clusters (i.e. non-orthodox Protestant individuals).

To determine differences in seroprevalences between males and females, between orthodox Protestant individuals and non-orthodox Protestant individuals and between both serosurveys first the parameters of the beta distribution for both seroprevalences were estimated using the methods of moments [24]. Next, risk ratios with their corresponding 95% confidence intervals (CIs) and p-values were estimated using Monte Carlo simulations of both serosurveys. Differences in GMCs between males and females and between both serosurveys were determined by testing the calculated difference between natural log transformed diphtheria antibody concentrations using the t-test.

#### Persistence of diphtheria antibodies (in NS)

Linear regression analysis was performed to assess the persistence of diphtheria antibodies in individuals from the NS completely immunized against diphtheria according to the NIP (i.e. with six diphtheria containing vaccinations), without self-reported or documented evidence of revaccination. To make results comparable to the 1995/1996 serosurvey, oversampled non-Western migrants from the 2006/2007 serosurvey were excluded from this analysis. Individuals who reported to be vaccinated because of profession were excluded from the analyses, as this indicates revaccination. The association between natural log transformed diphtheria antibody concentration and natural log transformed age was as such restricted to individuals who received the sixth vaccination at eight to nine years of age. Only individuals of 10 to 34 (1995/1996 serosurvey) [14] and 10 to 39 (2006/2007 serosurvey) years of age were included in the analyses. Older individuals were born before the current vaccination strategy. Individuals of 40 to 44 years of age were excluded from the analysis of the 2006/2007 serosurvey because this age group consisted only of three individuals. The lines fitting the data were based on the average of the log transformed diphtheria antibody concentrations per five-year age category. The difference between the lines from both serosurveys was determined using the F-test. To determine differences in proportions of individuals with antibody levels below 0.01 IU/ml between both serosurveys first the parameters of the beta distribution for both seroprevalences were estimated using the methods of moments [24]. Next, risk ratios with their corresponding 95% CIs and p-values were estimated using Monte Carlo simulations of both serosurveys.

#### Risk factors associated with diphtheria antibody levels below 0.01 IU/ml (in NS)

Logistic regression analysis was performed to assess odds ratios (ORs) and 95% CIs for possible risk factors associated with antibody levels below the minimum protective level of 0.01 IU/ml among individuals in the NS. Factors studied were: age; sex; religion; educational level (for children 14 years and younger the mothers’ highest educational level was asked for); number of registered diphtheria containing vaccinations; reported travel to high-risk regions (referring to travel to countries for which a DT-IPV-booster dose is advised); reported revaccination because of profession; ethnicity; net monthly income per household; degree of urbanization; geographical region; and years between last diphtheria containing vaccination (reported and registered) and blood sampling. For individuals aged 14 years and younger the last vaccination was based on the last vaccination given in the routine NIP since revaccination because of travel and/or profession is recommended 10 years after the last vaccination (i.e. at nine years of age). The same was done for those individuals aged 15 years and older that did not report revaccination. For individuals aged 15 years and older who reported revaccination, the time since the last revaccination was used.

Variables with a p-value <0.05 in the crude model adjusted for age and gender were included in the multivariable model. Backward selection was used to identify risk factors independently associated with antibody levels below 0.01 IU/ml.

## Results

In the 2006/2007 serosurvey 19781 individuals from the NS and 4366 individuals from the LVC sample were invited for study participation. Blood samples for diphtheria and questionnaire data were available for 6383 of the 6386 participants from the NS, including 645 individuals from the oversampled non-Western migrants, and for all 1518 participants from the LVC sample. In the 1995/1996 serosurvey 15189 individuals from the NS and 3028 individuals from the LVC sample were invited for study participation. Blood samples and questionnaire data were available for 7691 of the 8359 participants from the NS (no oversampling of non-Western migrants) and for 1492 of the 1589 participants from the LVC sample.

### Seroprevalence and GMC (in NS)

**Fig 1:**
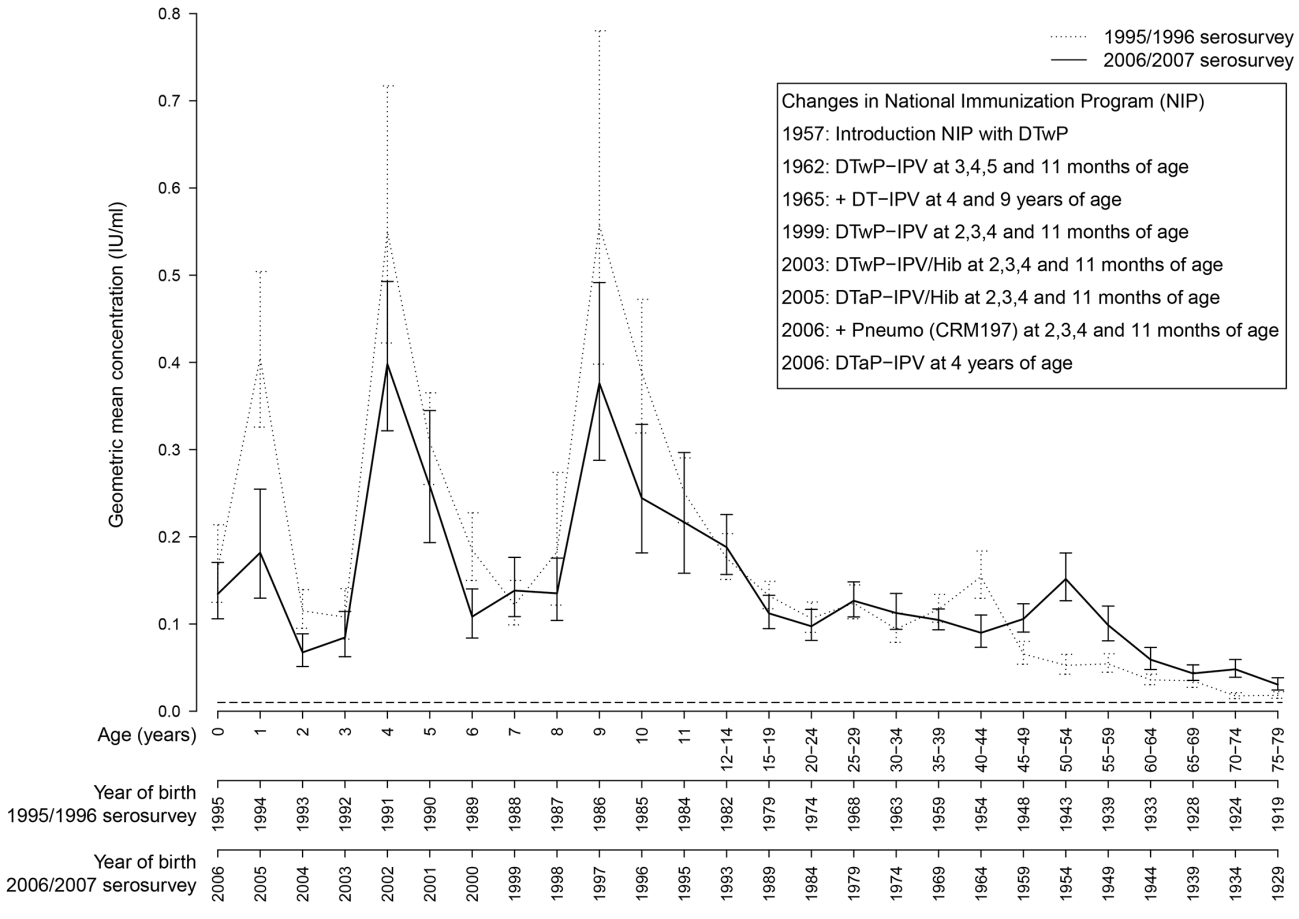
Weighted age-specific geometric mean IgG concentrations of diphtheria antibody in the national sample of the 1995/1996 serosurvey (n = 7691) and 2006/2007 serosurvey (n = 6383). The error bars represent the 95% confidence intervals. Year of birth indicates median year of birth corresponding to the defined age category. The dashed horizontal line represents the minimum level of protection of 0.01 IU/ml. The textbox shows changes in the National Immunization Program.

**Fig 2:**
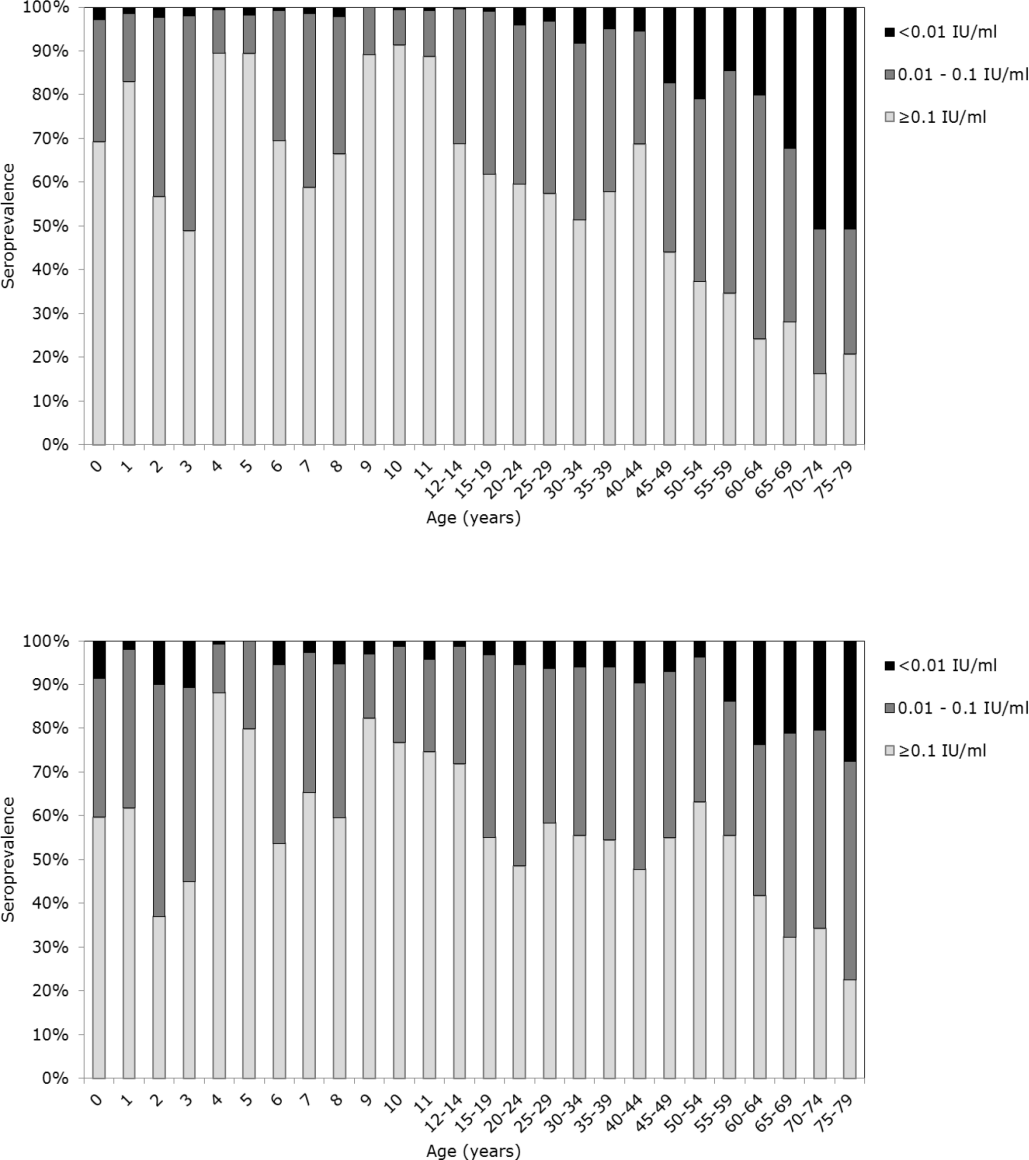
Weighted age-specific seroprevalences (%) of diphtheria antibody in the national sample of the 1995/1996 serosurvey (n = 7691) (Fig 2a) and in the national sample of the 2006/2007 serosurvey (n = 6383) (Fig 2b).

The proportion of individuals from the NS with antibody levels above the minimum protective level of 0.01 IU/ml was higher compared to the 1995/1996 serosurvey (90.6% vs. 88.4%, p = 0.001) (Table 1), with a corresponding higher GMC (0.10 vs. 0.09 IU/ml, p = 0.006). In the 2006/2007 serosurvey the proportion of males with antibody levels above 0.01 IU/ml was higher compared to females (92.9% vs. 88.3%, p<0.0001), also with a corresponding higher GMC (0.13 vs. 0.09 IU/ml, p<0.0001). The same result was found in the 1995/1996 serosurvey, where 91.2% of males and 85.6% of females (p<0.0001) had antibody levels above 0.01 IU/ml. GMCs for men and women were respectively 0.11 and 0.07 IU/ml (p<0.0001). Rises in the GMCs at one-, four- and nine years of age indicate the positive effect of vaccination (Fig 1). Geometric mean antibody concentrations declined rapidly after vaccinations, but stayed above 0.01 IU/ml in all age groups. The proportion of individuals who were born before introduction of diphtheria vaccination in the Netherlands (i.e. individuals of 40- and 51 years and older in the respectively 1995/1996 and 2006/2007 serosurvey) with antibody levels above 0.01 IU/ml was higher in the latter study (77.7% vs. 82.0%, p = 0.004) (Fig 2).

**Table 1:** Weighted seroprevalences (%) and geometric mean IgG concentrations (GMCs) of diphtheria antibody in the national sample of the 1995/1996 serosurvey (n = 7691) and 2006/2007 serosurvey (n = 6383).

	N	<0.01 IU/ml (%)	(95% CI)	0.01–0.1 IU/ml (%)	(95% CI)	≥ 0.1 IU/ml (%)	(95% CI)	GMC	(95% CI)
1995/1996 serosurvey									
Overall	7691	11.6	(10.7–12.6)	36.1	(34.4–37.9)	52.2	(50.4–54.1)	0.09	(0.09–0.10)
Men	3629	8.8	(7.7–9.9)	33.8	(31.5–36.1)	57.4	(54.6–60.2)	0.11	(0.10–0.12)
Women	4062	14.4	(13.1–15.8)	38.5	(36.5–40.5)	47.1	(44.9–49.2)	0.07	(0.07–0.08)
2006/2007 serosurvey									
Overall	6383	9.4	(8.4–10.3)	37.0	(35.7–38.2)	53.7	(52.0–55.4)	0.10	(0.10–0.11)
Men	2911	7.1	(5.9–8.3)	33.9	(32.1–35.8)	59.0	(56.9–61.1)	0.13	(0.12–0.13)
Women	3472	11.7	(10.4–13.0)	40.0	(38.0–41.9)	48.3	(46.0–50.7)	0.09	(0.08–0.09)

The increases in GMC at one- (p<0.0001), four- (p = 0.05) and nine- (p = 0.06) years of age were lower compared to the 1995/1996 serosurvey (Fig 1). Consistent with the 11-year period between both studies, the rise in GMC for 40 to 44-year-olds in the 1995/1996 serosurvey was observed among 51 to 55-year-olds in the 2006/2007 serosurvey.

### Seroprevalence and GMC (in LVC)

The proportion of individuals from the LVC with antibody levels above 0.01 IU/ml was 70.9% (95% CI 63.3–78.6) (Table 2). The proportion of non-orthodox Protestant individuals with protective antibody levels was higher compared to orthodox Protestant individuals (81.1% vs. 46.3%, p<0.0001). This was true for all age groups, except individuals of 65 years and older. Here the level of protection was comparable among orthodox Protestants, non-orthodox Protestants and individuals from the NS.

**Table 2:** Weighted seroprevalences (%) and geometric mean IgG concentrations (GMCs) of diphtheria antibody in orthodox Protestant individuals and non-orthodox Protestant individuals in the low vaccination coverage sample of the 1995/1996 serosurvey (n = 1492) and 2006/2007 serosurvey (n = 1518).

	N	<0.01 IU/ml (%)	(95% CI)	0.01–0.1 IU/ml (%)	(95% CI)	≥ 0.1 IU/ml (%)	(95% CI)	GMC	(95% CI)
1995/1996 serosurvey									
Overall	1492	25.4	(21.3–29.4)	30.7	(26.8–34.6)	44.0	(39.3–48.6)	0.06	(0.05–0.07)
Orthodox Protestant	233	62.9	(52.6–73.3)	15.6	(9.7–21.6)	21.5	(13.9–29.0)	0.01	(0.01–0.02)
Non-orthodox Protestant	1259	18.6	(15.8–21.5)	33.4	(29.8–36.9)	48.0	(43.0–53.0)	0.07	(0.06–0.08)
2006/2007 serosurvey									
Overall	1518	29.1	(21.4–36.7)	28.2	(24.6–31.9)	42.7	(34.4–51.0)	0.05	(0.04–0.08)
Orthodox Protestant	480	53.7	(45.0–62.5)	23.2	(17.8–28.5)	23.1	(19.1–27.1)	0.02	(0.02–0.03)
Non-orthodox Protestant	1038	18.9	(13.8–24.0)	30.3	(25.0–35.7)	50.8	(43.6–57.9)	0.08	(0.06–0.11)

A higher proportion of orthodox Protestants in the 2006/2007 serosurvey had antibody levels above 0.01 IU/ml compared to the 1995/1996 serosurvey (46.3% vs. 37.1%, p = 0.11).

### Persistence of diphtheria antibodies (in NS)

**Fig 3:**
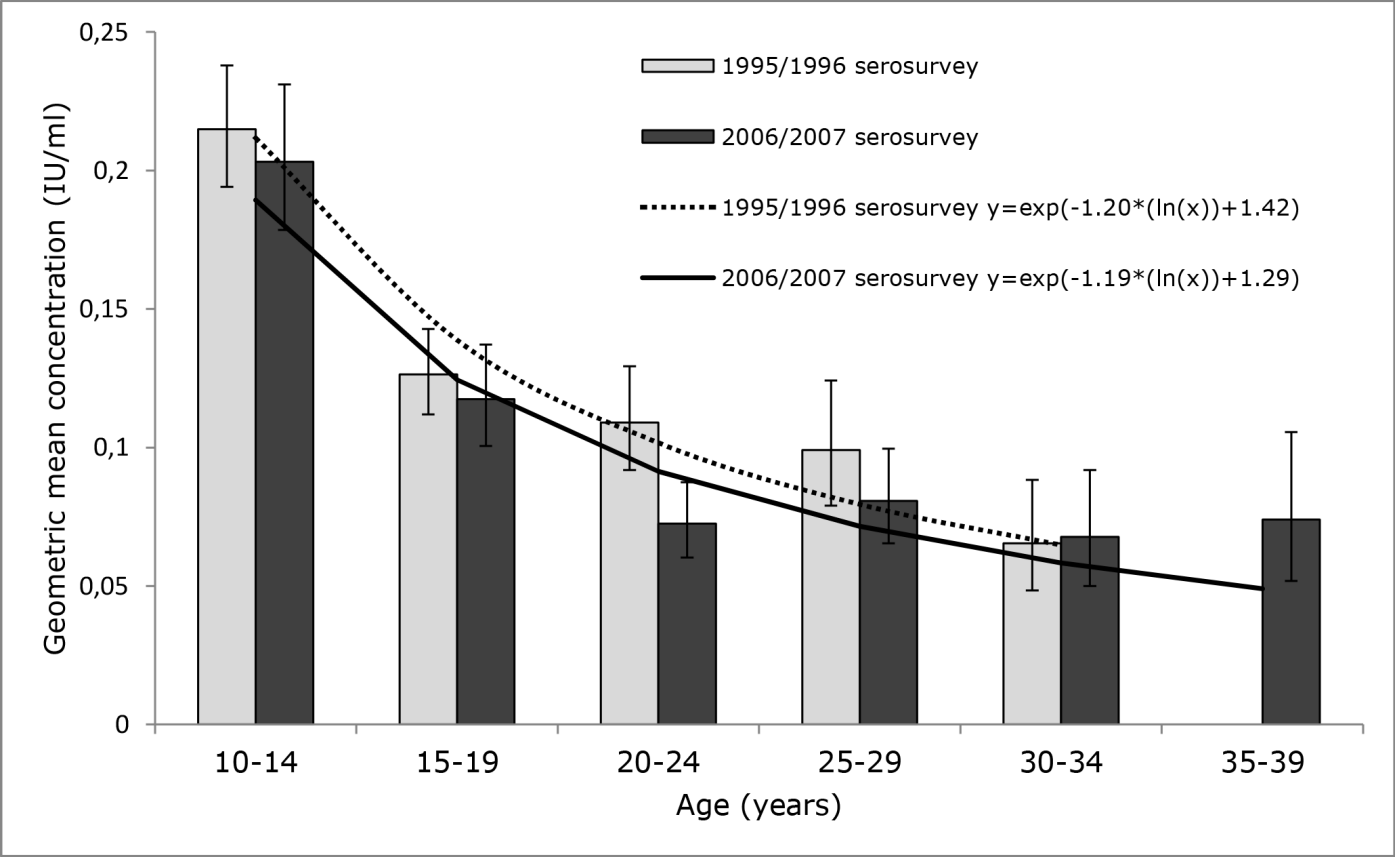
Persistence of diphtheria IgG antibody in 10 to 34 and 10 to 39 year old individuals, in the national sample of the 1995/1996 serosurvey (n = 961) and 2006/2007 serosurvey (n = 971), who were completely immunized against diphtheria according to the NIP, without evidence of revaccination.

A decline in antibody concentration with age was observed in 10 to 34- (1995/1996 serosurvey) and 10 to 39- (2006/2007 serosurvey) year-old individuals from the NS who received six diphtheria containing vaccinations according to the NIP (n = 961 and n = 971, respectively). The association between natural log transformed diphtheria antibody concentration and natural log transformed age indicated that diphtheria antibody concentrations declined at a comparable rate of -1.20 ln IU/ml per ln year and -1.19 ln IU/ml per ln year, respectively (p = 0.12) (Fig 3). Interpretation of the equation (example 2006/2007 serosurvey, for someone of 37 years of age): diphtheria antibody concentration = exp(-1.19*(ln(37))+1.29) = 0.05 IU/ml. GMCs remained well above 0.01 IU/ml for the oldest completely vaccinated age groups in both serosurveys (30 to 34 years of age and 35 to 39 years of age, respectively). In total 34 individuals (3.5%) from this specific cohort of the 2006/2007 serosurvey had antibody levels below 0.01 IU/ml, compared to eight individuals (0.8%) in the 1995/1996 serosurvey (p<0.0001) (Table 3). Of these, nine individuals had antibody levels below 0.01 IU/ml within 10 years after completing the NIP (i.e. 10 to 19-year-old individuals) in the 2006/2007 serosurvey, compared to one individual in the 1995/1996 serosurvey (p = 0.002). Note that cross-sectional data were interpreted longitudinally for the analyses of persistence of diphtheria antibodies in both studies.

**Table 3:** Age-specific seroprevalences (%) and geometric mean IgG concentrations (GMCs) of diphtheria antibody in 10 to 34 and 10 to 39 year old individuals, in the national sample of the 1995/1996 serosurvey (n = 961) and 2006/2007 serosurvey (n = 971), who were completely immunized against diphtheria according to the NIP, without evidence of revaccination.

Age (years)	N	<0.01 IU/ml (%)	(95% CI)	0.01–0.1 IU/ml (%)	(95% CI)	≥ 0.1 IU/ml (%)	(95% CI)	GMC	(95% CI)
1995/1996 serosurvey									
Overall	961	0.8	(0.3–1.4)	35.4	(32.4–38.4)	63.8	(60.7–66.8)	0.14	(0.14–0.16)
10–14	392	0.0	—	23.2	(19.0–27.4)	76.8	(72.6–81.0)	0.21	(0.19–0.24)
15–19	282	0.4	(0.0–1.0)	42.6	(36.8–48.3)	57.1	(51.3–62.9)	0.13	(0.11–0.14)
20–24	155	1.3	(0.0–3.1)	39.4	(31.7–47.1)	59.4	(51.6–67.1)	0.11	(0.09–0.13)
25–29	80	2.5	(0.0–5.9)	45.0	(34.1–55.9)	52.5	(41.5–63.5)	0.10	(0.08–0.12)
30–34	52	5.8	(0.0–12.1)	61.5	(48.3–74.8)	32.7	(19.9–45.5)	0.07	(0.05–0.09)
2006/2007 serosurvey									
Overall	971	3.5	(2.3–4.7)	40.3	(37.2–43.4)	56.2	(53.1–59.4)	0.12	(0.11–0.13)
10–14	346	1.2	(0.03–2.3)	25.1	(20.6–29.7)	73.7	(69.1–78.3)	0.20	(0.18–0.23)
15–19	217	2.3	(0.3–4.3)	41.9	(35.4–48.5)	55.8	(49.1–62.4)	0.12	(0.10–0.14)
20–24	180	6.1	(2.6–9.6)	53.3	(46.0–60.6)	40.6	(33.4–47.7)	0.07	(0.06–0.09)
25–29	119	5.0	(1.1–9.0)	49.6	(40.6–58.8)	45.4	(36.4–54.3)	0.08	(0.07–0.10)
30–34	72	8.3	(1.9–14.7)	51.4	(39.8–63.0)	40.3	(28.9–51.6)	0.07	(0.05–0.09)
35–39	37	5.4	(0.0–12.7)	56.8	(40.8–72.7)	37.8	(22.2–53.5)	0.07	(0.05–0.11)

### Risk factors associated with diphtheria antibody levels below 0.01 IU/ml (in NS)

In the univariable model degree of urbanization and geographical region were not statistically significantly associated with diphtheria antibody levels below 0.01 IU/ml. In the multivariable model, due to the inclusion of years between last diphtheria vaccination and blood sampling and number of registered diphtheria vaccinations, age group five to nine years and older age groups compared to age group zero to four years were negatively statistically significant associated with diphtheria antibody levels below 0.01 IU/ml (Table 4). In the multivariable model female compared to male, orthodox Protestant compared to non-orthodox Protestant, and middle-, and unknown education compared to high education were positively statistically significant associated with diphtheria antibody levels below 0.01 IU/ml. In addition, more than one year compared to less than one year between last diphtheria vaccination and blood sampling, having zero to one diphtheria vaccination compared to six diphtheria vaccinations, and no reported travel compared to reported travel to high-risk regions were positively statistically significant associated with diphtheria antibody levels below 0.01 IU/ml.

**Table 4:** Potential risk factors for having diphtheria antibody levels below 0.01 IU/ml in the national sample of the 2006/2007 serosurvey (n = 6383).

Potential risk factor	Categories	n (%)	Crude OR (95% CI)^a^	Adjusted OR (95% CI)
**Age group**	0–4	860 (13.5)	Ref	Ref
	5–9	620 (9.7)	0.5 (0.3–0.9)	0.4 (0.3–0.8)
	10–29	1441 (22.6)	0.6 (0.4–0.9)	0.3 (0.1–0.5)
	30–49	1356 (21.2)	1.1 (0.8–1.6)	0.2 (0.1–0.4)
	50–64	1130 (17.7)	2.4 (1.7–3.3)	0.3 (0.2–0.6)
	65–79	976 (15.3)	4.1 (3.0–5.7)	0.5 (0.3–0.8)
**Sex**	Male	2911 (45.6)	Ref	Ref
	Female	3472 (54.4)	1.7 (1.4–2.0)	1.5 (1.3–1.8)
**Religion**	Non-orthodox Protestant	6250 (97.9)	Ref	Ref
	Orthodox Protestant	133 (2.1)	2.9 (1.8–4.6)	2.2 (1.3–3.5)
**Educational level**	High	2401 (37.6)	Ref	Ref
	Middle	3137 (49.2)	1.5 (1.2–1.8)	1.3 (1.0–1.6)
	Low	730 (11.4)	1.4 (1.1–1.9)	0.9 (0.7–1.3)
	Unknown	115 (1.8)	2.6 (1.5–4.6)	2.0 (1.1–3.6)
**Years since last diphtheria vaccination**	0 (<12 months)	856 (13.4)	Ref	Ref
	1–4	1615 (25.3)	1.7 (1.0–2.8)	1.9 (1.1–3.1)
	5–9	728 (11.4)	2.7 (1.4–5.1)	2.8 (1.4–5.3)
	10–14	483 (7.6)	4.8 (2.5–9.1)	4.5 (2.4–8.7)
	15–19	297 (4.7)	6.1 (3.1–12.0)	4.8 (2.4–9.5)
	≥ 20	1836 (28.8)	9.2 (5.3–16.0)	5.7 (3.3–10.1)
	Not vaccinated	568 (8.9)	17.8 (10.3–30.9)	8.9 (5.0–15.8)
**Number of registered diphtheria containing vaccinations**	6	1578 (24.7)	Ref	Ref
	2–5	1959 (30.7)	0.6 (0.4–1.0)	0.8 (0.5–1.3)
	≥ 7	358 (5.6)	0.2 (0.1–0.7)	0.4 (0.1–1.0)
	0–1	2488 (39.0)	3.3 (2.3–4.7)	2.7 (1.9–3.9)
**Reported travel to high-risk regions**	Yes	2430 (38.1)	Ref	Ref
	No	3850 (60.3)	2.1 (1.7–2.5)	1.3 (1.1–1.7)
	Unknown	103 (1.6)	2.0 (1.1–3.7)	1.0 (0.5–1.9)
**Reported revaccination because of profession**	Yes	1077 (16.9)	Ref	
	No	3248 (50.9)	1.5 (1.2–1.9)	
	Unknown	2058 (32.2)	1.2 (0.8–1.8)	
**Ethnicity**	Dutch	4870 (76.3)	Ref	
	First generation other Western	153 (2.4)	1.7 (1.1–2.7)	
	Second generation other Western	292 (4.6)	0.8 (0.5–1.2)	
	First generation Turkey or Morocco	215 (3.4)	1.1 (0.7–1.9)	
	Second generation Turkey or Morocco	129 (2.0)	0.8 (0.4–1.9)	
	First generation Surinam, Aruba or Netherlands-Antilles	219 (3.4)	0.8 (0.5–1.2)	
	Second generation Surinam, Aruba or Netherlands-Antilles	138 (2.2)	0.2 (0.1–0.9)	
	First generation other non-Western	230 (3.6)	1.4 (0.9–2.1)	
	Second generation other non-Western	137 (2.2)	0.8 (0.4–1.7)	
**Net monthly income per household**	High (≥ € 3051,-)	1087 (17.0)	Ref	
	Middle (€ 1151,- - € 3050,-)	2950 (46.2)	1.5 (1.2–2.1)	
	Low (≤ 1150,-)	1004 (15.7)	2.0 (1.5–2.8)	
	Did not want to answer	1110 (17.4)	1.9 (1.4–2.6)	
	Unknown	232 (3.6)	1.8 (1.1–3.0)	

^a^ Adjusted for age and gender

## Discussion

The results of both population-based serosurveillances studies indicated that the general Dutch population was well protected against diphtheria. However, 18% of individuals who were born before introduction of diphtheria vaccination in the NIP (i.e. individuals of 51 years and older) had antibody levels below the minimum protective level of 0.01 IU/ml. In addition, 54% of social and geographical clustered orthodox Protestants who refuse vaccination on religious grounds lack adequate levels of diphtheria antibodies.

Overall, the results compare well with the transformed results of the 1995/1996 serosurvey and are in between findings from other European countries [25–28]. The most remarkable differences between both serosurveys were the lower GMCs until 11 years of age (i.e. high antibody levels due to vaccination) in the 2006/2007 serosurvey. In particular for the one-year-olds, impact of changes in schedule and vaccine source might be a possible explanation. Infants in the 1995/1996 serosurvey received the vaccinations at three, four, and five months of age compared to two, three, and four months of age for infants in the 2006/2007 serosurvey. We found the first peak in antibody levels however at respectively six and four months of age (data not shown). Thus, starting vaccination at a later age might have been associated with higher responses to vaccination, which has also been reported before [29–31]. In 2005 the infant whole-cell pertussis vaccine was replaced by an acellular pertussis vaccine. Miller and colleagues [32], in contrast to Pichichero [33] found lower immunogenicity of the diphtheria component in the combination vaccine with acellular pertussis. Furthermore, infants in the 1995/1996 serosurvey received the DTPw-IPV vaccine^®^ from the National Institute for Public Health and the Environment (RIVM) and separately Hib, while infants in the 2006/2007 serosurvey received either the combination vaccine Infanrix IPV+Hib^®^ from GSK or Pediacel^®^ from SP MSD. Thus, our results suggest that Infanrix IPV+Hib^®^ and Pediacel^®^ do not induce an immune response as high as the DTPw-IPV vaccine^®^.

A third national seroepidemiological study (Pienter3) that is planned for 2016/2017 could give further insight into the (course of) antibody levels in these cohorts.

In both the 1995/1996 serosurvey and the 2006/2007 serosurvey we found a rise in GMC among those born between 1952 and 1956 (i.e. aged 40 to 44 years and 51 to 55 years respectively). Individuals born just before the introduction of routine vaccination in 1957 might have received more, or at an older age, diphtheria vaccinations than individuals born after introduction of routine vaccination as it was noted that children were already widely vaccinated at school going age, before the introduction of the NIP in 1957 [34].

Among orthodox Protestants aged 65 years and older similar levels of diphtheria antibodies were found as among non-orthodox Protestants and participants in the NS aged 65 years and older. This reflects the similar natural exposure to *Corynebacterium diphtheria* before introduction of diphtheria vaccination.

A higher proportion of orthodox Protestants had antibody levels above 0.01 IU/ml compared to the 1995/1996 serosurvey. Since natural exposure to *Corynebacterium diphtheriae* rarely exists in the Netherlands since 1960, we expect that a higher proportion of orthodox Protestant individuals received vaccinations in 2006/2007.

This is in line with a study of Ruijs and colleagues who found a movement towards higher acceptance of vaccination among the majority of orthodox Protestants, except among the most conservative denominations (personal communication Helma Ruijs 29 April 2015, National Institute for Public Health and the Environment). Nevertheless, in 2006/2007 still 54% of the socio-geographically clustered orthodox Protestants had antibody levels below 0.01 IU/ml. From 1997 to 2014 only five solitary (imported) diphtheria cases were reported [10], which implies that herd immunity in the Netherlands is sufficient.

Females had a higher risk of having diphtheria antibody levels below 0.01 IU/ml compared to males, which was already reported by others [27,35,36]. A possible explanation might be that men were more likely to have received booster doses during military service [14] or for other profession.

We calculated that among completely immunized individuals of the 2006/2007 serosurvey a statistical significantly higher proportion of individuals had antibody levels below 0.01 IU/ml within 10 years after completing the NIP compared to the 1995/1996 serosurvey. We have no clear explanation for this increase. The diphtheria containing vaccines used in both periods for the birth cohorts included in this analysis were from the National Vaccine Institute [37] and the bacterium has not been circulating in both periods. Military service including DT-IPV vaccination was compulsory until 1996. Therefore, perhaps more individuals in the 1995/1996 serosurvey received vaccinations due to the military service. We adjusted for this by excluding individuals who reported to be vaccinated because of profession. However, it might be that more revaccinated individuals were included in the analysis of the 1995/1996 serosurvey compared to the 2006/2007 serosurvey. When we analyzed the data for women only the difference was not statistical significant (p = 0.09) anymore.

We acknowledge that a limitation of our study is the use of two different assay techniques. The assay used for the 2006/2007 serosurvey was a MIA while for the 1995/1996 serosurvey a different test, i.e. a ToBI, was used. We have taken this into account by using a correction factor to enable bridging between both serosurveys. It seems therefore unlikely that the use of two different assay techniques can explain for the differences in antibody concentrations observed between the two serosurveys. Furthermore, both ToBI [38] and MIA [39] performed good in two consecutive external quality assessment studies (EQA) where the assays were compared to the Vero cell neutralization test (NT), a test considered as the *in vitro* gold standard.

The participation rate was smaller compared to the 1995/1996 serosurvey (32% vs. 55%, respectively), so response bias might be present in our study. However, for most important factors we corrected using weights.

The study has several important strengths. It is a large population-based study. It was possible to do subgroup analyses and extensive information is available for most participants. The serosurveys were conducted with an 11-year interval making it possible to do a comprehensive comparison of antibody levels by age in time.

In conclusion, the NIP provides long-term protection against diphtheria, although antibody levels decline after vaccination. As a result of natural waning immunity, a substantial proportion of individuals born before introduction of diphtheria vaccination lack adequate levels of diphtheria antibodies. Susceptibility due to lack of vaccination is highest among strictly orthodox Protestants.

The potential of importation of diphtheria cases remains, as diphtheria is still endemic in some countries. Therefore, the threat of spread of diphtheria within the geographically clustered orthodox Protestant community has not yet disappeared, despite national overall long-term high vaccination coverage.

## Supporting Information

S1 File Data underlying the equation which was used to transform all concentrations of the 1995/1996 serosurvey measured with ToBI to make them comparable for the 1995/1996- and 2006/2007 serosurveys and data underlying the Bland-Altman plot. The file includes the comparison of diphtheria antibody concentrations (IU/ml) as measured by the ToBI and MIA and the Bland-Altman plot.
(XLSX)

S2 File Data underlying all tables and figures of this article are available in the S2 File. (XLS)
